# Verification of *Arabidopsis* stock collections using SNPmatch, a tool for genotyping high-plexed samples

**DOI:** 10.1038/sdata.2017.184

**Published:** 2017-12-19

**Authors:** Rahul Pisupati, Ilka Reichardt, Ümit Seren, Pamela Korte, Viktoria Nizhynska, Envel Kerdaffrec, Kristina Uzunova, Fernando A. Rabanal, Daniele L. Filiault, Magnus Nordborg

**Affiliations:** 1 Gregor Mendel Institute (GMI), Austrian Academy of Sciences, Vienna Biocenter (VBC), Dr. Bohr-Gasse 3, Vienna 1030, Austria; 2 Insitute of Molecular Pathology (IMP), Vienna Biocenter (VBC), Campus-Vienna-Biocenter 1, 1030 Vienna, Austria; 3 Vienna Graduate School of Population Genetics, Institut fÃ¼r Populationsgenetik, Vetmeduni, Vienna, Austria

**Keywords:** Genomics, Natural variation in plants

## Abstract

Large-scale studies such as the *Arabidopsis thaliana* ‘1,001 Genomes’ Project require routine genotyping of stocks to avoid sample contamination. To genotype samples efficiently and economically, sequencing must be inexpensive and data processing simple. Here we present SNPmatch, a tool that identifies strains (or inbred lines, or accessions) by matching them to a SNP database. We tested the tool by performing low-coverage resequencing of over 2,000 strains from our lab seed stock collection. SNPmatch correctly genotyped samples from 1-fold coverage sequencing data, and could also identify the parents of F1 or F2 individuals. SNPmatch can be run either on the command line or through AraGeno (https://arageno.gmi.oeaw.ac.at), a web interface that permits sample genotyping from a user-uploaded VCF or BED file.

## Introduction

Sample contamination is an unavoidable problem when large-scale experiments are performed. For example, cell lines are frequently misidentified or contaminated, and the need for validation has long been underappreciated^1,2^. The same problem applies to germplasm collections such as plant seed resources of elite cultivars and crops^3^. These collections are expanding rapidly with the increasing number of experiments utilizing natural variation. Recently, the genomes of 1,135 *Arabidopsis thaliana* strains were sequenced^4^ and this panel (hereinafter referred to as ‘1,001 Genomes’ panel) is now widely used. The need for verifying seed stocks is clear^5^. Routine quality checks of seed stock genotypes can guard against common mistakes such as tube mislabeling or seed contamination during harvesting. In principle, genotyping can be easily performed by short-read sequencing due to its high throughput and low error rates. Sequencing and library preparation costs are dropping rapidly, both for reduced-representation methods like restriction-site-associated-DNA sequencing (RAD-seq) as well as whole-genome sequencing^6,7^. In contrast, user-friendly tools for the analysis of sequencing data are only starting to become available.

Here we present SNPmatch, a simple tool for efficiently identifying strains by matching them to a database of strain genotypes. SNPmatch implements a likelihood model to identify matching strains for a given set of markers (SNPs) in the individual. We validated SNPmatch using published sequences of the *A. thaliana* ‘1,001 Genomes’ panel^4^. SNPmatch readily identified correct genotypes with only a few thousand random SNP markers—numbers easily achieved by any sequencing effort. We then used SNPmatch to perform a quality check of a lab seed stock collection. We performed inexpensive, low-coverage sequencing of the seed stock collection by multi-plexing 192 libraries into a single Illumina sequencing lane, resulting in a median sequencing coverage of 1.8X per sample. Using SNPmatch, a staggering 10% of our stocks were found to be mis-identified. These mistakes are currently being investigated and remedied. This result clearly demonstrates the need for, and utility of, quality control by sequencing.

SNPmatch is a Python library which can be run on the command line, and we also developed AraGeno (https://arageno.gmi.oeaw.ac.at), a simple web interface that allows users to query their own SNP data against the public *A. thaliana* ‘1,001 Genomes’ polymorphism databases.

## Results

### Validating SNPmatch using the ‘1001 Genomes’ of *A. thaliana*

We validated SNPmatch using the data from the published ‘1,001 Genomes’ of *A. thaliana*^4^. First, we used raw sequencing reads to investigate how our ability to genotype samples depends on sequencing depth. We thinned the data to one, three, and six million reads for each sample to test the effect of coverage (one million reads roughly corresponds to multiplexing 192 *A. thaliana* samples in a single Illumina Hi-Seq 2,500 lane, or roughly 2X coverage) (Data Citation 1). The ability to genotype samples was essentially unaffected by this level of thinning, indicating that even 192-fold multiplexing would yield sufficient SNPs for SNPmatch to accurately perform strain identification (Supplementary Tables 1 and 2).

**Figure 1:**
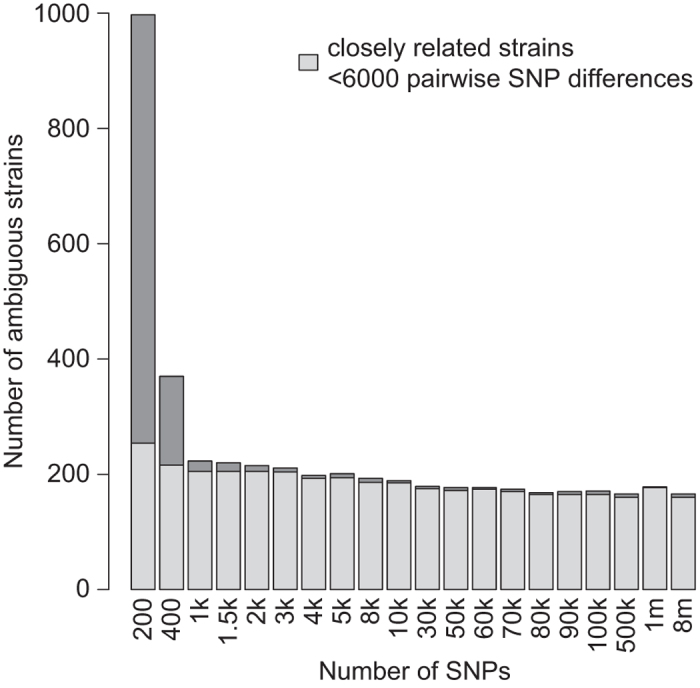
Validation of SNPmatch. Number of samples ambiguously identified by SNPmatch given a random subsets of SNPs from the published ‘1,001 Genomes’ dataset. Almost all of the strains which were ambiguously matched have less than 6,000 pairwise differences to at least one other strain (highlighted in blue).

We next investigated the number of SNPs required to unambiguously genotype individual strains. Using the published SNP calls of the ‘1,001 Genomes’ panel (Data Citation 2), we randomly selected subsets of SNPs and ran SNPmatch using only these subsets. As illustrated in Fig. 1, the number of strains uniquely identified is largely independent of the number of SNPs, provided that this number exceeds a few thousand and as long as the strains are not very closely related In the ‘1,001 Genomes’ panel, there are 79 North American strains and 63 other pairs of strains that differ by less than 6,000 SNPs (Supplementary Fig. 5). These strains are difficult to distinguish from each other even with millions of SNPs (Fig. 1) or few millions of reads (Supplementary Table 1). Smaller sets of SNPs are unlikely to include informative markers.

### Verification of our lab seed stock collection using SNPmatch

**Figure 2:**
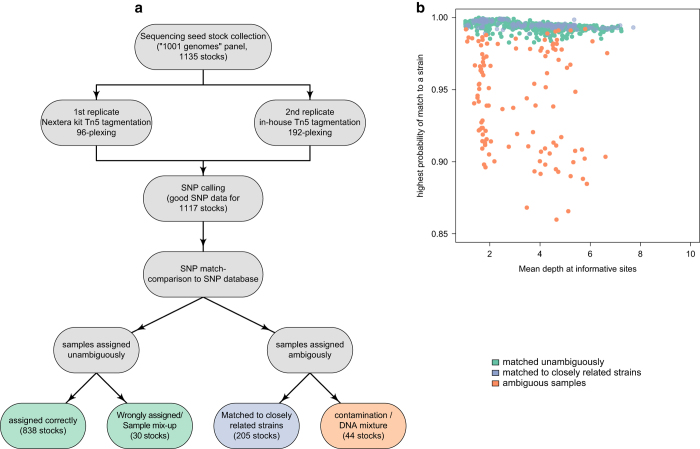
Validation of seed stock collection using SNPmatch. (**a**) Schematic workflow for the validation of the lab ‘1,001 Genomes’ seed stock collection. (**b**) Genotyping results for all resequenced stocks in at least two replicates. SNPmatch efficiently genotyped samples regardless of the sequencing depth.

In order to verify our lab seed stocks of the ‘1,001 Genomes’ panel^4^, we resequenced a nearly-complete set of this collection (Data Citation 3). We performed SNP calling on all samples as detailed in Methods and ran SNPmatch using a SNPmatch database constructed from published data of the 1,001 Genomes Project (Fig. 2a). As expected, the sequencing depth did not affect genotyping performance (Fig. 2b and Supplementary Table 2).

Of a total of 1,135 stocks belonging to the ‘1,001 Genomes’ collection, 1,117 yielded more than 2,000 SNPs necessary for analysis. Of these, 1,043 were assigned to the correct strain (or a set of strains differing by less than 6,000 SNPs in the database). Of the remaining 74 stocks, 30 stocks were unambiguously assigned to the wrong strain (or set of closely related strains), indicating stocks or strains mix-identified (Supplementary Table 3). The remaining 44 stocks did not match any strain in the database. These stocks could represent unknown strains, DNA contamination, or outcrossed individuals. However, the sequencing data of the non-matching stocks do not contain an unusually large number of heterozygous calls, thus, outcrossing or DNA contamination is unlikely (Supplementary Fig. 6; see also our analysis of F2 individuals). Furthermore, DNA contamination can be excluded since biological replicates produced identical results. Therefore, we conclude that these 44 stocks are most likely due to stocks having been replaced by unknown strains. We are currently collecting and verifying the 74 incorrect stocks from different sources in order to ensure that the stock center has the right germplasm. So far, 49 out of 74 incorrect stocks have been correctly identified and will be replaced (Supplementary Table 3).

We are currently verifying our stocks for a previously-released *A. thaliana* stock collection called the ‘RegMap’ panel^8^. We will provide updates on these analyses on the 1,001 Genomes website (http://1001genomes.org) as they become available.

### Identifying parents of hybrid individuals using SNPmatch

SNPmatch can also be used to identify hybrid individuals when parental strains are present in the database. To demonstrate this, we generated two hybrid (F2s) *A. thaliana* populations (Fäb-2 x Stenk-3 and Gro-3 x ÖMö2-1). We sequenced 96 F2 individuals for each cross (Data Citation 4), performed SNP calling and filtering as detailed in Methods below, and ran the SNPmatch algorithm. The parental lines were drawn from the ‘RegMap’ stock collection^8^. Therefore, we used database files constructed from the published ‘RegMap’ collection SNP data (Data Citation 2) as detailed in Methods.

**Figure 3:**
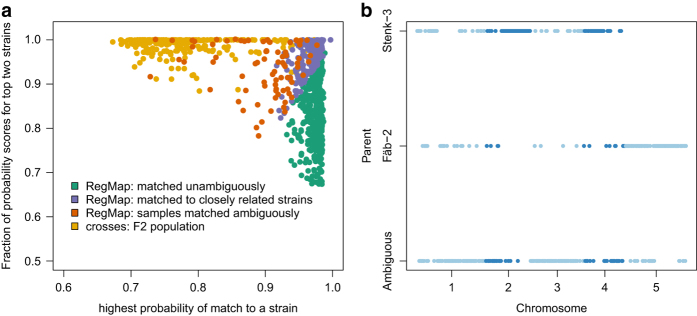
SNPmatch on hybrid population and identifying parents. (**a**) Distinguishing hybrid population (F2s) and samples in the ‘RegMap’ panel using SNPmatch. (**b**) A cross (Fäb-2 x Stenk-3) was analysed using SNPmatch in 300 kb windows. Each blue dot (Chr 1, 3 and 5 in light blue and Chr 2 and 4 in dark blue) represents a window across the genome matching either unambiguously to one of the parents or ambiguously (y-axis).

As expected, F2 individuals show a large fraction of heterozygous calls (Supplementary Fig. 6), and do not unambiguously match one strain in the database, but rather closely and equally to both parents (Fig. 3a). For such individuals, running SNPmatch in windows across the genome quickly reveals their hybrid nature (Fig. 3b).

### AraGeno

**Figure 4:**
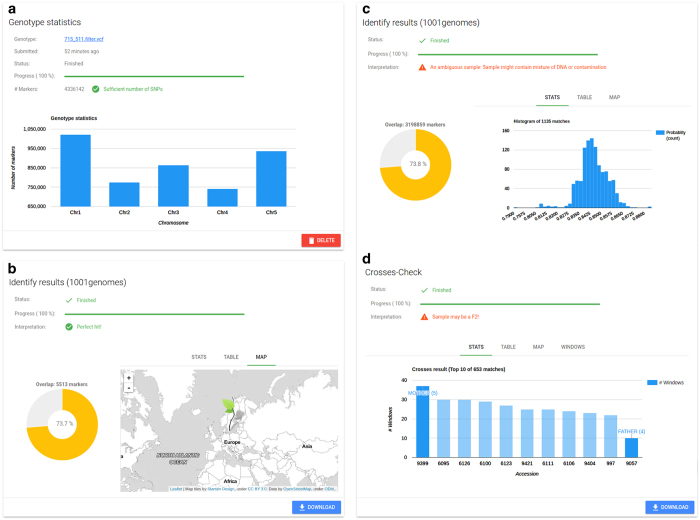
AraGeno web-application. (**a**) General information about the uploaded genotype (VCF or BED) as well as the status of the parsing is displayed. (**b**,**c**) The results of the SNPmatch analysis are displayed in separate panels for two major SNP databases (‘1,001 Genomes’ and ‘RegMap’). A pie-chart highlights the overlap between the user-provided genotype and the SNP database. Depending on the analysis result, either a histogram or a column chart is displayed of the top matches. A table and map provide additional information about the highest matched strains. (**d**) If SNPmatch detects that the sample is possibly an individual of an F2 individual, an additional analysis starts automatically and, in case the cross is confirmed, information about the parents are displayed.

SNPmatch is a Python library primarily intended to be used as a command line tool. We ensured that the installation of SNPmatch is as simple and straightforward as possible by hosting SNPmatch on the official Python software repository PyPi (https://pypi.python.org/pypi). Nevertheless, we aimed to go a step further and also provide SNPmatch as a service to the *Arabidopsis* community by taking care of the installation process and data handling. To this end, we developed AraGeno (https://arageno.gmi.oeaw.ac.at), which provides a web-based frontend to SNPmatch. Users will only need to provide a VCF or BED file, while AraGeno validates the data running SNPmatch on our servers, and a summary of the analysis will be displayed in an interactive web-interface (Fig. 4).

Our system can easily process many analyses concurrently. Furthermore, AraGeno provides a RESTful API for programmatic access to the SNPmatch pipeline. AraGeno is a Python based web-application using the Django framework (https://www.djangoproject.com/) and Django REST framework (http://www.django-rest-framework.org/) for the backend and Vue.js (https://vuejs.org/) for the interactive front end side.

### SNPmatch, tool to validate germplasm collections

We developed SNPmatch that can be easily implemented to other species and to validate their germplasm collections. We have also tested SNPmatch on Medicago truncatula inbred lines sequenced under ‘HapMap’ project^9^. Using publicly available polymorphism databases (Data Citation 6), few thousands of randomly selected SNPs are sufficient to identify the correct genotype among 262 available strains (Supplementary Fig. 7). Since the *M. truncatula* ‘HapMap’ collection does not contain very closely related strains, all the strains were uniquely identified with just a couple of thousand SNPs.

## Discussion

With rapid growth in public germplasm resources, strain identification is an increasingly key part of maintaining stocks available to the entire scientific community. Two web tools for genotyping *A. thaliana* strains have been developed: ANATool^10^ and StrainID (http://tools.1001genomes.org/strain_id/). However, neither tool is capable of handling many samples at a time, and the analyses performed by both tools are limited to their own pre-defined sets of *A. thaliana* strains. Hence we developed SNPmatch, a python package for high-throughput genotyping of samples from the sequencing data. SNPmatch is ideally suited to high-throughput analyses given its speed and input data format. Additionally, when users run SNPmatch on the command line, they can construct and use their own SNPmatch databases, thereby extending their analyses to any relevant *A. thaliana* germline panel (as demonstrated in our F2 analyses), or even potentially to genotyping other species.

Two additional characteristics of the SNPmatch algorithm make it an ideal tool for strain identification. First, the likelihood model used by SNPmatch provides a statistical estimate for the best match, providing users a measure of certainty of strain identification. Secondly, SNPmatch can genotype samples using extremely sparse SNP marker data, permitting strain identification with economical highly-multiplexed resequencing data. Additionally, SNPmatch can also be used with SNPs generated from transcriptome or bisulfite sequencing data, making it ideal for routine checks of strain identification in many types of large-scale experiments. Although the number of markers required will depend on the population structure of the database strains used, in the ‘1,001 Genomes’ panel of *A. thaliana*, for example, 930 strains can be distinguished from each other using 2,000 SNPs (Fig. 1). The remaining 205 strains which differ by fewer than 6,000 pairwise SNPs from another strain or strains (Supplementary Fig. 5) would likely either require targeted sequencing of segregating sites or the use of molecular methods such as DNA fingerprinting.

SNPmatch can be implemented to any strain identification problem, a valid question in inbreeding species or landraces. While we have tested SNPmatch thoroughly on our *A. thaliana* seed stocks, there is also the possibility to use SNPmatch to genotype stocks of other species such as *Medicago truncatula*, *Lotus japonicus* and landraces of Rice and Maize. In *Medicago truncatula*, SNPmatch identified strains readily with only few thousand SNPs (Supplementary Fig. 7). However, assumption of binomial distribution in the likelihood model makes SNPmatch restricted to diploid species with biallelic markers. But, SNPmatch can be easily adapted for polyploids by changing the likelihood model.

Finally, SNPmatch is an user-friendly reliable tool for genotyping samples with minimal number of SNPs and can be routinely used for validating germplasm collections. Also SNPmatch is implemented as a simple web tool, AraGeno, for *A. thaliana* users.

## Methods

### Plant samples

About five gas-sterilised seeds of each stock of the entire ‘1,001 Genomes’ and ‘RegMap’ lab seed stock collections were sown directly on soil and stratified for 6 days at 4 °C in the dark. Plants were grown at 10 °C under long days (16 h light, 8 h dark) with 60% humidity. DNA was extracted from 2–3 leaves using the NucleoMag Plant Kit (Macherey Nagel, cat. #744400).

Segregating populations used in this manuscript are derived from crosses between Fäb-2 (6,917; CS28247) and Stenk-3 (9,454; CS77275) and between Gro-3 (6,025; CS76889) and ÖMö2-1 (7,518; CS22584). Numbers in parenthesis indicate unique ecotype IDs used in the framework of the ‘1,001 Genomes’ Project^4^ and stock center IDs (CS), respectively. For each population, 96 individuals were grown at 10 °C under long days (16 h light, 8 h dark) with 60% humidity. DNA was isolated from flower buds using the NucleoMag Plant Kit (Macherey Nagel, cat. #744400).

### Library preparation and sequencing

In this study we used two different methods for library preparation. The first method follows the commercial Illumina Nextera library preparation protocol modified by Baym and colleagues^11^. For the second method we assembled transposomes from Tn5 transposase (Lucigen). The genotyping results using the two library preparation methods are comparable (Supplementary Table 2).

For the first round of sequencing (1st replicate of the ‘1,001 Genomes’ collection+‘RegMap’ panel) and for the F2 individuals, libraries were prepared as described by Baym *et al.*^11^. Briefly, 2.5 ng of DNA was tagmented and adapter ligated in a 2.5 μl reaction volume using Illumina NexteraTM Kit. Libraries were amplified by Illumina TrueSeq Primers and VeraSeq High Fidelity DNA Polymerase (Biozym, cat #280390). Size selection and PCR clean-up was performed with Agencourt AMPure Beads (Beckman Coulter, cat. #A63882). Libraries were validated with Fragment AnalyzerTM Automated CE System (Advanced Analytical) pooled in equimolar concentration for 96-multiplex. Libraries were sequenced on Illumina HiSeqTM 2,500 Analyzers using manufacturers standard cluster generation and sequencing protocols in 125 bp PE mode.

For the second round of sequencing (2nd replicate of the ‘1,001 Genomes’ collection) we used Tn5 transposase from Lucigen. Transposome assembly was performed as described in Picelli *et al.*,^12^. Briefly, it was made from a 0.143 vol of 100 μM equimolar mixture of preannealed Tn5MEDS-A and Tn5MEDS-B adapters and diluted 20 times using a dilution buffer (50 mM Tris-HCl pH 7.5, 100 mM NaCl, 0.1 mM EDTA, 50% glycerol, 0.1% Triton X-100, 1 mM DTT). The diluted Tn5 stock was incubated for 30 min at 37 °C. Assembled Tn5 was stored at −20 °C and used for up to two months. Newly assembled Tn5 was tested on different DNA concentrations each time before usage. Tn5 reactions were assembled in small volumes (2.5 μl total) by mixing 0.5 μl TAPS buffer (50 mM TAPS-NaOH pH 8.5, 25 mM MgCl2, 50% DMF), 0.1 μl of preassembled Tn5, 1.0 μl DNA (2.5 ngμl^−1^) and 0.9 μl H2O. Reactions were incubated for 7 min at 55 °C. Tagmented DNA was used directly for PCR amplification. PCR was performed by mixing 2.5 ng/μl of tagmented DNA with 11.2 μl VeraSeq High Fidelity DNA Polymerase (Biozym, cat #280390) and 8.8 μl of 0.5 μM index oligos. The PCR program was as follow: 3 min 72 °C, 5 min 98 °C, 8–14 cycles of 10 s 98 °C, 30 s 63 °C, 30 s 72 °C, and 5 min 72 °C. PCR reactions were purified by adding 23 μl AMPure XP beads (cat. # A63882, Beckman Coulter) to 22.5 μl PCR volume. DNA was eluted in 25 μl H2O. Libraries were validated with Fragment AnalyzerTM Automated CE System (Advanced Analytical) and pooled in equimolar concentration for 192-multiplex. Libraries were sequenced on Illumina HiSeqTM 2,500 Analyzers using manufacturers standard cluster generation and sequencing protocols in 50 bp paired-end mode.

### Sequencing data analysis

Sequencing data for inbred lines (Data Citation 3) and F2 individuals (Data Citation 4) were demultiplexed by custom scripts and quality checked using FastQC^13^. Reads were aligned to the *A. thaliana* reference genome (TAIR10, https://www.arabidopsis.org/index.jsp) using bwa mem (version 0.7.13)^14^ with default parameters. Next, duplicates were marked and removed using picard tools software suite^15^ using default parameters. SNP and indel calls were obtained from alignment files using GATK HaplotypeCaller (version 3.5)^16^ following the Best Practice instructions (https://software.broadinstitute.org/gatk/best-practices/, accessed April, 2017) on joint genotyping for all the samples per plate. Briefly, we used HaplotypeCaller to generate GVCF (genomic variant calling file) on each sample individually. Subsequently we implemented GenotypeGVCFs, a join genotyping tool from GATK, together on all the generated GVCFs for either 96 or 192 samples based on the plexing level of sequencing. Finally, we filtered for biallelic SNPs using ‘Select Variants’ option of GATK. All the above GATK scripts were run with the default parameters.

### SNPmatch algorithm

The input to SNPmatch is SNPs in a variant calling format file (VCF file). SNPmatch then performs the following calculations to identify the most likely strain from a database.

1. For each database strain ‘a’, SNPmatch calculates the probability of the sample matching to it, *p*_*a*_. This is calculated from the genotype probability scores (PL) from GATK if present.

$$p_{a}=\sum_{j}P\left(g_{j}|a_{j}==g_{j}\right)$$

where,

*j* is the the genomic position of the marker, *a*_*j*_ is the genotype of database strain ‘a’ at position *j* and *g*_*j*_ is the genotype of sample at position *j*. This is one of the outputs from SNPmatch, and is referred to as the probability of match to a strain.

2. Likelihoods are calculated for each strain based on the binomial distribution.

$$L_{a}=(p_{a}\times n_{a}\times ln(p_{a}/(1-p)))+((1-p_{a})\times n_{a}\times ln((1-p_{a})/p))$$

where,

*n*_*a*_ is the number of informative sites between strain a and the sample, *p* is the error rate (0.001).

3. Likelihood ratios (LR) are calculated for each strain with the top likely strain.

$$LR_{a}=L_{a}/\max(\{L_{i}:i=1,..,n\})$$

under the assumption that LR is a chi-squared distribution, a threshold of 3.841 gives a list of strains that are not significantly different from the top hit (at a 95% confidence level).

### Code availability

Both the source code for SNPmatch and AraGeno are hosted on GitHub (https://github.com/Gregor-Mendel-Institute/SNPmatch, https://github.com/Gregor-Mendel-Institute/AraGeno).

### Quick guide for running SNPmatch

#### Installing SNPmatch

SNPmatch can be easily installed with pip (https://github.com/pypa/pip). All the required dependencies should be installed automatically. Detailed instructions are given in the github repository (https://github.com/Gregor-Mendel-Institute/SNPmatch#installation-using-pip).

> pip install SNPmatch

Successful installation can be tested with the presence of ‘snpmatch’ bash executable in PATH bash environment. This can be checked by running ‘snpmatch -h’, resulting in a detailed description of available commands.

#### Preparing database files

Database files contains strain genotype information against which samples are compared. These are required for the SNPmatch analysis and can be generated from a VCF file using the commands below. The database file can have any number of biallelic markers with any number of strains merged into one single file (input_vcf). The VCF file should contain the genotype data in ‘GT’ tag indicating the two alleles carried by each strain. The allele values are 0 for reference allele and 1 for the alternative allele, separated by either ‘/’ or ‘|’ for biallelic position.

> snpmatch makedb -i input_vcf -o db

This generates two files ‘db.hdf5’ and ‘db.acc.hdf5’ used in the following analysis. We have generated database files for the published biallelic SNP data in ‘1,001 Genomes’ and ‘RegMap’ panels (Data Citation 2). The genotype information in these panels is for 1,135 and 1,307 strains with 10.7 million and 214 thousand informative positions respectively. When running SNPmatch to validate ‘1,001 Genomes’ or ‘RegMap’ seed stocks, we compared resequenced samples to database files of the corresponding panel.

We have also generated database files for published biallelic SNP data in ‘HapMap’ project of *Medicago truncatula* (Data Citation 2) using above command. To investigate the genotyping ability of SNPmatch in *M. truncatula*, we have used these database files and ran SNPmatch on randomly selected subsets of SNPs.

#### Usage

SNPmatch can be run as an executable bash command. The database and sample genotype files are given as inputs. These are detailed on the README file of Github repository (https://github.com/Gregor-Mendel-Institute/SNPmatch/blob/master/README.md). In short, SNPmatch is a single command as shown below for a given ‘input_file’.

> snpmatch inbred -d db.hdf5 -e db.acc.hdf5 -i input_file \-o output_file

To run the algorithm in windows, as was done in analysis of F2 populations, the user can choose the ‘window_size_in_bp’ option as given below.

> snpmatch cross -d db.hdf5 -e db.acc.hdf5 -i input_file \-b window_size_in_bp -o output_file

The output of the above command is the genotyping result on each window of given size across the genome. Users interested in applying SNPmatch to other species are advised to change a global variable, ‘chrlen’, in csmatch script under core folder in SNPmatch (https://github.com/Gregor-Mendel-Institute/SNPmatch/blob/master/snpmatch/core/csmatch.py#L19). This variable corresponds to chromosome length of the species, presently set to the chromosome lengths of the *A. thaliana* TAIR 10 genome.

### Data availability

The re-sequencing data for the almost complete ‘1,001 Genomes’ and ‘RegMap’ panels has been submitted to NCBI Bioproject accession number SRP100124 (Data Citation 3). An expandable interactive table is found in NCBI RunSelector (https://www.ncbi.nlm.nih.gov/Traces/study/?acc=SRP100124). The low-coverage sequencing of two *A. thaliana* F2 populations (Fäb-2 x Stenk-3 and Gro-3 x ÖMö2-1) is published in NCBI Sequence Read Archive accession SRP102034 (Data Citation 4). The SNP datasets used for *A. thaliana* ‘1001 Genomes’, ‘RegMap’ panel are uploaded in figshare (Data Citation 2). The SNP datasets for *M. truncatula* ‘HapMap’ collection were also generated as described in Methods and uploaded in figshare (Data Citation 2).

## Additional information

**How to cite this article:** Pisupati, R. *et al.* Verification of *Arabidopsis* stock collections using SNPmatch, a tool for genotyping high-plexed samples. *Sci. Data* 4:170184 doi: 10.1038/sdata.2017.184 (2017).

**Publisher’s note:** Springer Nature remains neutral with regard to jurisdictional claims in published maps and institutional affiliations.

## Supplementary Material

Supplementary Information
