# Supplementary material for: Verification of Arabidopsis stock collections using SNPmatch, a tool for genotyping high-plexed samples
Source: Sci Data. 2017 Dec 19;4:170184. doi: 10.1038/sdata.2017.184 (PMC5744633; doi:10.1038/sdata.2017.184)
Supplement: Supplementary Information [file sdata2017184-s1.pdf]

Supplementary material

Verification of *Arabidopsis* stock collections using SNPmatch, an algorithm for genotyping high-plexed samples

Rahul Pisupati, Ilka Reichardt, Ümit Seren, Pamela Korte, Viktoria Nizhynska, Envel Kerdaffrec, Kristina Uzunova, Fernando Rabanal, Daniele Filiault and Magnus Nordborg*

Supplementary Figure S1 1

Supplementary Figure S2 2

Supplementary Figure S3 2

Supplementary Table S1 2

Supplementary Table S2 3

Supplementary Table S3 3

Supplementary Figure S1

Pairwise comparisons. Histogram of pairwise single nucleotide differences between strains in the "1001 Genomes" panel. The panel above is zoomed in for x-axis range from zero to eight thousand pairwise differences.

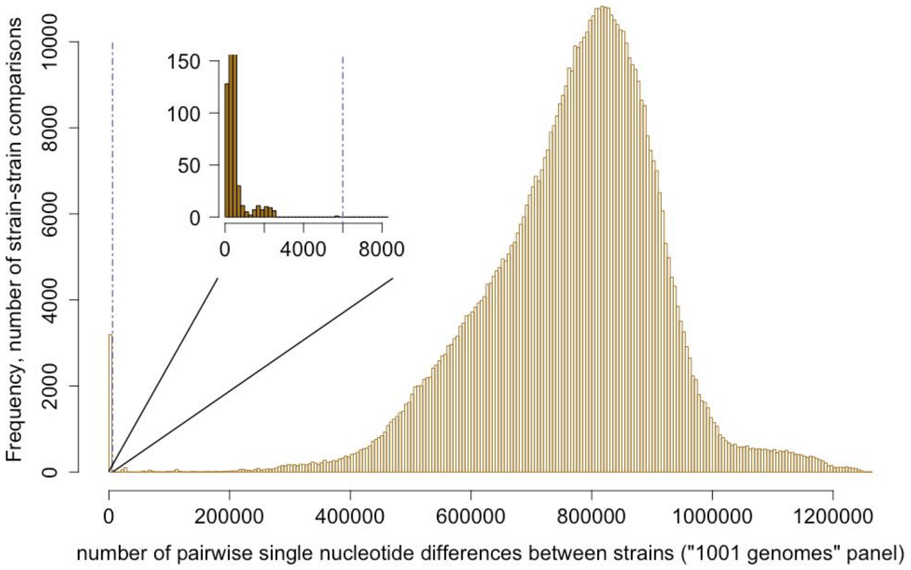

Supplementary Figure S2

The fraction of heterozygous SNPs in samples that do not unambiguously match to any strain (red) are comparable to other samples. F2 individuals (orange) have a much higher fraction of heterozygous calls when their coverage is good.

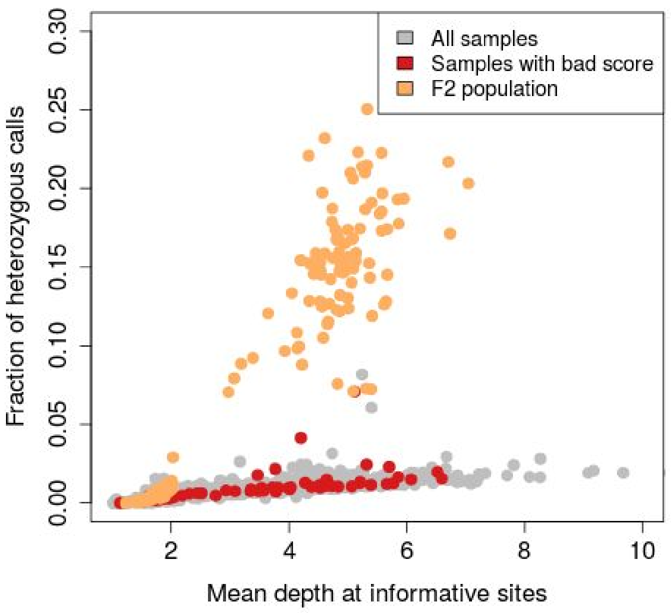

Supplementary Figure S3

Number of samples ambiguously identified by SNPmatch given random subsets of SNPs from the *Medicago truncatula* “HapMap” project (Mt 4.0 based). SNPmatch readily identified correct genotypes with only a few thousand random SNP markers.

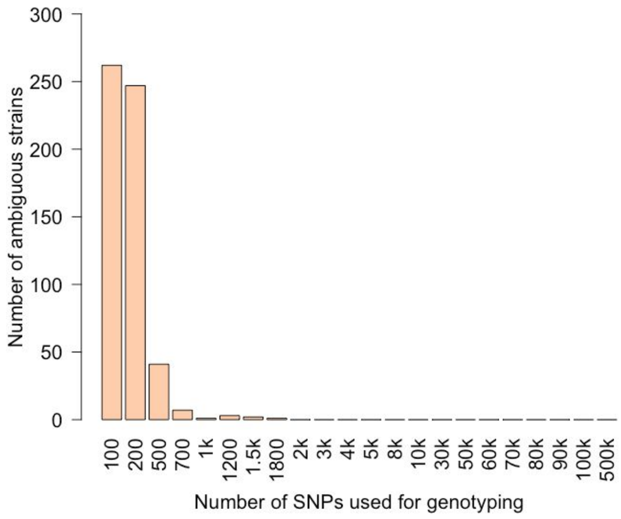

Supplementary Table S1

Genotyping results using SNPmatch after subsetting specific number of reads from published "1001 Genomes" raw data.

Number of reads	Total strains analysed	Unambiguously identified	% ambiguous
1 million	1110	914	17.66
3 million	1118	918	17.89
6 million	1117	912	18.35

Supplementary Table S2

SNPmatch results for the two replicates resequenced with different library preparation protocols.

Round	Library preparation protocol	Multiplexing per Illumina lane	Mean depth	% unambiguous samples	Mean probablity of match for unambiguous hits
1	Nextera kit Tn5 tagmentation	96	4.67	79%	0.993
2	Lucigen Tn5 tagmentation	192	1.9	80.40%	0.997

Supplementary Table S3

List of strains in "1001 genomes" panel which needed attention. We identified the correct origin for some of the strains (colored in orange) and the correct seeds will be replaced. The origin of for strains colored in red are still unknown.

incorrectly assigned strains				
Ecotype ID	name	stock center ID	origin	top hit
8387	St-0	CS77270	SWE	8386
7250	Me-0	CS76549	GER	7394

9314	Gol-2	CS76883	UK	9937
9437	Puk-2	CS77195	SWE	9436
9503	11C1	CS76640	UK	9910
9622	Bijisk-4	CS76707	RUS	9737
9632	Lebja-2	CS77016	RUS	9642
9642	Rakit-3	CS77204	RUS	9632
9658	Nicas-1	CS77127	ITA	9655
7125	Er-0	CS78898	GER	9761
9761	Bik-1	CS76449	LBN	9764
9790	Gn2-3	CS76881	GER	9777
9908	ESP-1-11	CS76847	FRA	9314
9910	BRI-2	CS76725	FRA	9503
7307	Pn-0	CS77182	FRA	7316
9912	CIRY-13	CS76773	FRA	6909
7418	Zu-1	CS78881	SUI	6744
9927	ARR-17	CS76673	FRA	9935
9928	BEZ-9	CS76703	FRA	9908
7566	627ME-13Y1	CS78367	USA	2159
9935	BAU-15	CS76694	FRA	9927
6680	ANH-1	CS76437	GER	6990
7063	Can-0	CS76740	ESP	7186
9312	Ullapool-8	CS78821	UK	9912
7138	Fi-0	CS76488	GER	7125
9739	Toc-1	CS77370	ROU	9738
9911	ARGE-1-15	CS76672	FRA	9928
9914	IST-29	CS76948	FRA	9312/9917
9929	ISS-20	CS76947	FRA	9925
1819	Lak-12	CS78974	USA	6909

strains that don't match anything

Ecotype ID	name	stock center	origin

	ID		
9624	Chaba-2	CS76767	RUS
9646	Aiell-1	CS76648	ITA
9655	Marce-1	CS77071	ITA
9657	Melic-1	CS77078	ITA
9659	Pigna-1	CS77177	ITA
7417	Zu-0	CS78880	SUI
9691	Castelfed-3-20<br>7	CS76756	ITA
9697	Dolen-1	CS76802	BUL
9707	Podvi-1	CS77187	BUL
9709	Zerev-1-34	CS78878	BUL
9714	Grivo-1	CS76888	BUL
9716	Leska-1-44	CS77030	BUL
9723	Slavi-2	CS77252	BUL
6990	Amel-1	CS76434	NED
9729	Stiav-2	CS77280	SVK
9735	Bela-4	CS76699	SVK
9737	Ulies-1	CS78815	ROU
9741	Orast-1	CS77151	ROU
9747	Zabar-1	CS78870	SRB
7218	Le-0	CS76540	NED
7280	Old-1	CS76567	GER
9917	RAD-21	CS77200	FRA
7332	Seattle-0	CS76598	USA
8354	Per-1	CS76571	RUS
8420	Kelsterbach-4	CS76525	GER
9610	Lesno-4	CS77034	RUS
9615	Parti-1	CS77163	RUS
9937	CATS-6	CS76760	FRA
6198	TDr-13	CS77347	SWE
7427	Ko-2	CS76531	DEN
7164	Hau-0	CS76915	DEN

9592	IP-Vae-2	CS78827	ESP
9608	Karag-2	CS76961	RUS
9661	Cimin-1	CS76771	ITA
7377	Tul-0	CS76618	USA
7415	Wu-0	CS78858	GER
9909	GEN-8	CS76877	FRA
9915	Mar-4-16	CS77069	FRA
9727	Olympia-2	CS77144	GRC
9920	DIR-9	CS76796	FRA
9921	FOR-23	CS76867	FRA
9924	PLY-20	CS77181	FRA
9930	LEC-25	CS77018	FRA
9932	NOZ-6	CS77134	FRA
